# Modulation of Adipogenic Conditions for Prospective Use of hADSCs in Adipose Tissue Engineering

**DOI:** 10.3390/ijms131215881

**Published:** 2012-11-27

**Authors:** Bianca Galateanu, Sorina Dinescu, Anisoara Cimpean, Anca Dinischiotu, Marieta Costache

**Affiliations:** Department of Biochemistry and Molecular Biology, University of Bucharest, 91-95, Splaiul Independentei, sect. 5, Bucharest 050095, Romania; E-Mails: bianca.galateanu@gmail.com (B.G.); sorina_d31@yahoo.com (S.D.); anisoara.campean@gmail.com (A.C.); dinischiotu@yahoo.com (A.D.)

**Keywords:** lipoaspirate, hADSC, adipogenesis, PPARγ2, *perilipin*, *aP2*, *FAS*, Oil Red O

## Abstract

Modern strategies in adipose tissue engineering (ATE) take advantage of the easy harvest, abundance and differentiation potential towards mesenchymal lineages of hADSCs. The controlled conversion of hADSCs to committed adipogenic precursors and further mature adipocytes formation is important for good long-term results in soft tissue regeneration. Thus, in this study, we report: (i) the isolation of the processed lipoaspirate (PLA) cells from adipose tissue and sanguine fractions; (ii) the phenotypic characterization of the PLA descendants; (iii) the design of a novel protocol for the modulation of adipogenic conditions in the perspectives of ATE applications. To modulate the differentiation rate through our protocol, we propose to selectively modify the formulation of the adipogenic media in accordance with the evolution of the process. Therefore, we aimed to ensure the long-term proliferation of the precursor cells and to delay the late adipogenic events. The status of differentiation was characterized in terms of intracellular lipid accumulation and reorganization of the cytoskeleton simultaneously with perilipin protein expression. Moreover, we studied the sequential activation of *PPARγ2*, *FAS*, *aP2* and *perilipin* genes which influence the kinetics of the adipogenic process. The strategies developed in this work are the prerequisites for prospective 3D regenerative systems.

## 1. Introduction

Over the last decade, there has been a paradigm shift in the understanding of the nature of adipose tissue. For a long time, the white adipose tissue (WAT), derived from the mesenchyme, has been considered to be a passive organ for the storage of energy to be mobilized during food deprivation with the release of fatty acids for oxidation in other organs. However, research has demonstrated that WAT is a dynamic endocrine organ of considerable complexity, which is highly integrated into the overall physiologic and metabolic control systems of mammals [1]. Adipocytes actively secrete numerous hormones, growth factors and cytokines, such as leptin, adiponectin, tumor necrosis factor-α (TNF-α), interleukin-6 (IL-6) *etc.*, which influence energy homeostasis [2], sexual hormone synthesis, immunity, and also regulate the function of the circulatory system.

Recent studies [3] have shown that subcutaneous adipose tissue provides a clear advantage over other stem cell sources due to its accessibility, minimal morbidity and the discomfort associated with its harvest. Therefore WAT represents an attractive source of autologous adult stem cells for regenerative therapy due to its abundance, surgical accessibility, and high content of multipotent mesenchymal [4,5] and endothelial [6,7] progenitor cells. Thus, while for many years bone marrow-derived mesenchymal stem cells (BM-MSC) were the primary source of stem cells for tissue engineering applications, the stromal-vascular fraction (SVF) of adipose tissue consists of a heterogeneous cell population including endothelial precursor cells, preadipocytes, anti-inflammatory M2 macrophages, and mesenchymal stem cells (MSC), as well [8].

Due to their secretory profile, human adipose-derived stem cells (hADSCs), delivered into injured or diseased tissue stimulate recovery in a paracrine manner. These cells modulate the “stem cell niche” of the host by stimulating the recruitment of endogenous stem cells to the site of injury and promote their differentiation along the required lineage pathway [9]. hADSCs secrete nearly all of the growth factors that take part in normal wound healing [10–13]. After implantation, these cells may remain viable at the wound site and secrete growth factors in a continuous and regulated manner in response to environmental cues, just as it occurs in the natural wound healing process [14]. The hADSCs differentiation potential towards the mesenchymal lineages concomitant with materials science advances encouraged the design of hADSC-scaffold biohybrids for adipose tissue engineering (ATE) applications.

Rodbell *et al.*[15,16] presented the first methods to isolate cells from adipose tissue from minced rat fat pads. Subsequently, this procedure has been modified for the isolation of cells from human adipose tissue specimens [17,18]. Later on, Dominici *et al.*[19] described the multipotent status of the mesenchymal stromal cells, whereas Gimble *et al.*[9] published a review on the isolation, characterization and preclinical and clinical application of adipose-derived stem cells.

The term “adipose-derived stem cells” (ADSCs) was introduced by Zuk *et al.*[6] to show that SVF contains large numbers of MSC-like cells that could be induced to differentiate into adipogenic, chondrogenic, myogenic, and osteogenic lineages [6]. Further, after *in vitro* expansion, the same group [20] reported that SVF-derived cells expressed similar surface markers to BM-MSC, revealing the positive expression of CD29, CD44, CD71, CD90, CD105/SH2, and SH3 and lacking CD31, CD34, and CD45.

The differentiation of adipocytes from precursor stem cells involves a complex and highly orchestrated program [21]. The acquisition of the adipocyte phenotype is characterized by chronological changes, reflected in the activation of early, intermediate and late adipogenic specific markers. Overall, triglyceride accumulation, concomitant with dramatic changes that occur in cell morphology, cytoskeletal components and extracellular matrix are typical to different adipogenic stages.

Many genes are directly or indirectly involved in the regulation of adipogenesis in mammalian species and represent the critical markers for monitoring this process. The genes encoding transcription factors including PPAR (Peroxisome Proliferator-Activated Receptor) and C/EBP (CCAAT/enhancer binding protein) have been identified both *in vitro* and *in vivo*[22]. They directly influence fat cell development by encoding transcriptional factors that regulate gene expression of downstream genes typically involved in lipid and fatty acid metabolism. These factors do not work completely independently but interact functionally in several important ways.

PPARγ2 acts as the key inducer and master regulator of the adipogenic differentiation process [23]. Its activity is controlled by free fatty acids or by the thiazolidinediones antidiabetic drugs, such as troglitazone. Thus, PPARγ2 has been found to regulate the expression levels of many genes that are involved in adipocyte differentiation including adipocyte fatty acid–binding protein (aP2), lipoprotein lipase (LPL), fatty acid synthase (FAS), perilipin, fatty acid transport protein-1 (FATP-1), adiponectin or leptin, which are involved in lipid storage and the control of metabolism [24,25].

Once activated, *FAS*, *aP2* and *perilipin* act together in order to synthesize, transport and mediate triacylglycerols (TAG) metabolism, respectively. First, the synthesis of long-chain saturated fatty acids is catalyzed by *FAS*. Insulin and triiodothyronine (T3) interact synergistically and have positive effect on *FAS* gene transcription [26]. Next, aP2 works as a transmembrane carrier for newly synthesized fatty acids and allows their accumulation inside maturating adipocytes. The lipid accumulations are surrounded by a dynamic structure at the periphery, which controls the access of lipolytic enzymes to the fatty acids, as phosphorylated perilipin provides the docking site for the activated hormone-sensitive lipase (HSL) [27].

Although the sequence of events occurring during adipogenesis is already characterized in terms of molecular signaling, a more elaborate approach is a must in the prospective use of hADSCs in adipose tissue engineering (ATE). Modern strategies in current ATE applications involve the design of 3D cell-scaffold constructs. To achieve *in situ* functional *de novo* tissue, the undifferentiated cells implanted in a 3D scaffold are required to proliferate and to populate its entire volume. Once exposed to adipogenic induction *in vitro*, the differentiation kinetics must be proportionally connected with the scaffold’s degradation rate.

Considering our previous work regarding the proliferation and the differentiation potential of hADSCs in natural-based 3D hydrogels [28], in this study we intend to control the progress of the adipogenic events during differentiation in monolayer cultures in the view of prospective use in regenerative medicine studies based on 3D *in vitro* models. What is more, previous studies stated that *in vitro* adipogenic differentiation of preadipocytes varies with differentiation stimulus, culture dimensionality and scaffold composition and concluded that alterations in 3D scaffold physical properties significantly impacted the ability of preadipocytes to undergo adipogenic differentiation *in vitro*[29]. Although 3D culture systems are considered to be more appropriate for differentiation studies as they can closely reproduce the environment that cells experience *in vivo*, we decided to perform the modulation of the adipogenic conditions in a 2D system. This decision is a consequence of the high number of variables that 3D systems provide in terms of scaffold composition and structure, cell distribution and interactions that could influence the process of differentiation in contrast with 2D systems. Furthermore, 2D experimental models allow a much easier observation of cell behavior in contact with the adipogenic cocktail of inducers than in 3D approaches. This 2D approach offers the potential to reduce the number of experimental studies in more costly 3D culture systems.

Consequently, our research aimed to design a novel protocol for the modulation of adipogenic conditions in the perspective of selecting the appropriate adipogenic media formulation for *in vitro* ATE experiments in order to prolong the cellular proliferative state and to delay the late events of adipogenesis.

## 2. Results and Discussion

### 2.1. Primary Culture of PLAs-Derived Cells

**Figure 1:**
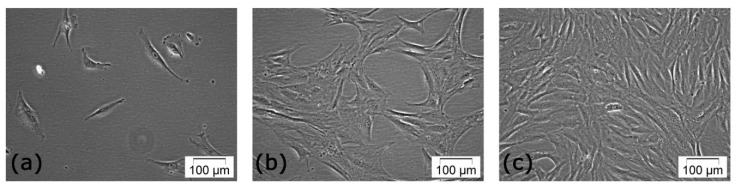
Phase contrast micrographs of cells in primary culture at: (**a**) 24 h; (**b**) 5 days and (**c**) 15 days post-seeding.

Both primary cultures derived from adipose tissue and sanguine fractions of the lipoaspirate (LA) were used in our studies. Following isolation and seeding, the heterogeneous cell suspension was microscopically inspected in phase contrast. The few cells remaining attached to the culture dishes after 24 h post seeding exhibited a fibroblast-like spindle shape. After three to four days, adherent isolated cells started to proliferate. At six to seven days, they formed small clusters and, after two weeks of incubation a confluent uniform monolayer was achieved. Occasionally, nonadherent round mononuclear cells were found in the primary culture, but they were removed during subcultivation (Figure 1).

No differences were observed in the morphological features of both primary cell cultures during subcultivation. In addition, extracting cells from both adipose tissue and sanguine fractions resulted in a higher efficiency in terms of amount of isolated cells, as compared to extracting cells from one single fraction.

As the cells were propagated in monolayer culture, they showed a more uniform fibroblast-like morphology, suggesting the existence of a homogeneous hADSC cell population.

### 2.2. Characterization of hADSC

#### 2.2.1. hADSCs Cell Surface Antigen Expression

Cells isolated from adipose tissue fraction of LAs in primary culture and their descendants obtained during the first ten passages of subcultivation were subjected to phenotypic characterization in order to establish the crucial passage when a pure hADSC population was achieved. Zuk *et al.*[5] demonstrated the presence of multiple CD marker antigens, similar to those observed on MSCs, on the surface of processed lipoaspirate (PLA) cells.

**Figure 2:**
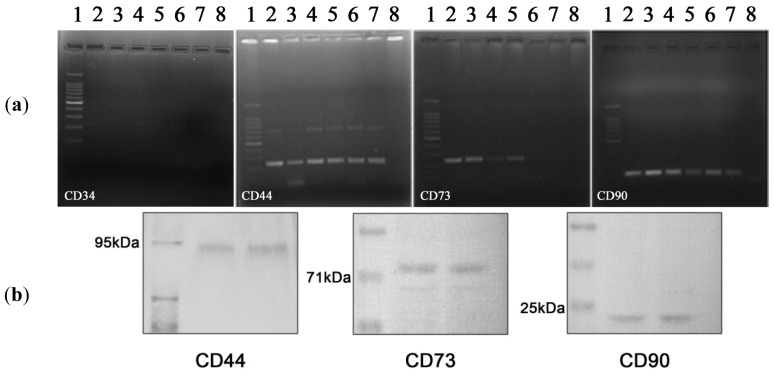

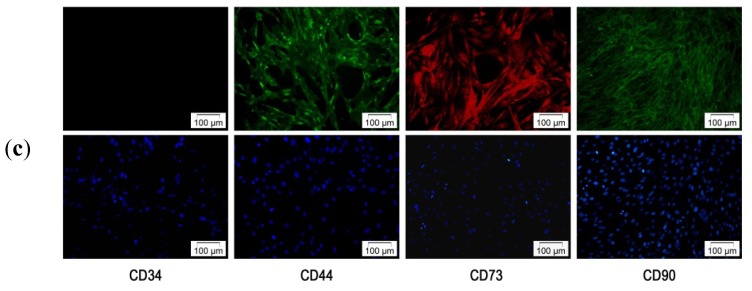
Phenotypic characterization of cultured hADSCs in third passage (**a**) Agarose gel electrophoresis of gradient RT-PCR products for CD34, CD44, CD73 and CD90; All surface markers were tested at 6 different temperatures in the range of 52–62 °C, as follows: (2) 54.9 °C; (3) 55.8 °C; (4) 56.6 °C; (5) 57.4 °C; (6) 58.3 °C; (7) 59.1 °C, while (1) corresponds to the molecular marker of 100 bp used for visualization and (8) represents the negative control (no template control); (**b**) Western blotting for duplicate detection of CD44, CD73 and CD90 in cell lysates; (**c**) Fluorescence micrographs revealing negative immunostaining for CD34 hematopoietic stem cell marker and positive labeling for CD44, CD73 and CD90 stromal stem cell surface antigens (upper panel) and cell density by nuclei-DAPI staining (lower panel).

Considering this, the surface phenotype of hADSCs was examined via quantitative q-PCR reaction. All PLA descendants were shown to be positive for stromal stem cell surface markers CD44, CD73 and CD90, as revealed by agarose electrophoresis of gradient RT-PCR products (Figure 2a). In addition, no expression of CD34 was observed starting with the third passage cells.

CD44, CD73 and CD90 antigens protein expression on the surface of hADSCs was confirmed by Western Blot analysis (Figure 2b). Moreover, hADSCs are negative for the hematopoietic marker CD34 starting with the third passage and positive for the stromal cell markers CD44, CD73 and CD90 as highlighted by immunofluorescence microscopy (Figure 2c).

All these data suggest that the third passage hADSCs express the markers associated with BM-MSCs. Thus, the pattern of surface antigens displayed by hADSCs in our study is in accordance with the phenotype of stem cells isolated from the bone marrow, umbilical-cord blood [30] and resembles with the results reported by Zuk *et al.*[20] and Gronthos *et al.*[4]. Consequently, only cells obtained starting with the third passage can be considered suitable PLA descendants for further adipogenic studies. Based on senescence studies we performed (data not shown), hADSCs between the third and the seventh passages proved to be appropriate for prospective investigations.

#### 2.2.2. Adipogenic Differentiation of hADSC

The differentiation potential of hADSC was extensively studied in the last decade [2,6,9,22,31]. It is already known that these cultured cells are able to undergo adipogenesis, chondrogenesis and osteogenesis in the presence of certain inducers. The adipogenic differentiation process differs by species, donor’s age, sex and tissue location [31] and thereby requires the adjustment of experimental parameters. However, for tissue engineering purposes, the differentiation process must be controlled in terms of clonal expansion rate and adipogenesis kinetics.

In our study, the adjustment of adipogenic differentiation was performed by means of adipogenic cocktail variation. Thereby, a set of main inducers consisting of 3-iso-butyl-1-methylxanthine (IBMX), dexamethasone (DEX) and troglitazone was established. IBMX is known to upregulate C/EBP, a transcriptional factor responsible for Krüppel-like transcription factor 5 (KLF5) activation. Subsequently, KLF5 triggers PPARγ2 promoting further transcription of the target genes [32]. The synthetic glucocorticoid DEX is a powerful adipogenic inducer for precursor cells isolated from different species, which probably acts by the inhibition of pref-1 expression [31]. DEX increases the C/EBP and PPARγ expression in 3T3-L1 preadipocytes. Tiazolidindione-induced adipogenesis was observed in multiple cell culture models since troglitazone, a PPARγ ligand, is a crucial adipogenic factor [31]. Insulin was a constant compound in the adipogenic cocktail. Although the amount of insulin receptors on the surface of progenitor cells is low, the insulin effect increases during the adipogenic process. Insulin promotes hADSCs differentiations by activating PI3-kinase and Akt-signaling pathways, which subsequently stimulate the expression of adipocyte specific genes, such as *FAS*, leptin and adiponectin [2].

Considering ATE as our final goal, four different protocols (P_0_, P_1_, P_2_ and P_3_) were proposed for the identification of an adequate adipogenic strategy. These protocols were created by mixing five different adipogenic media (MD_0_–MD_4_), as described in Tables 1 and 2 Taking into account the effect of each pro-adipogenic molecule, P_0_–P_3_ were designed for the administration of inducers separately from the differentiation supporting factors (biotin, indomethacine, hydrocortisone, T3 and transferrin). Moreover, insulin was added in all cocktails (MD_0_–MD_4_) and IBMX, DEX and troglitazone were considered as the main adipogenic inducers. Thus, P_1_ was based on the incubation of the hADSC monolayers with the most complex adipogenic cocktail (MD_1_). P_2_ consisted in the same strategy for the first three days of induction, followed by the exclusion of the main adipogenic inducers from the differentiation medium (MD_2_). To provide a slow cell differentiation, P_3_ was designed using the minimal set of pro-adipogenic molecules required for the activation and maintenance of the process (MD_3_, MD_4_). All these strategies were related to the use of a reference adipogenic medium (MD_0_), administered constantly for up to 21 days (P_0_).

**Figure 3:**
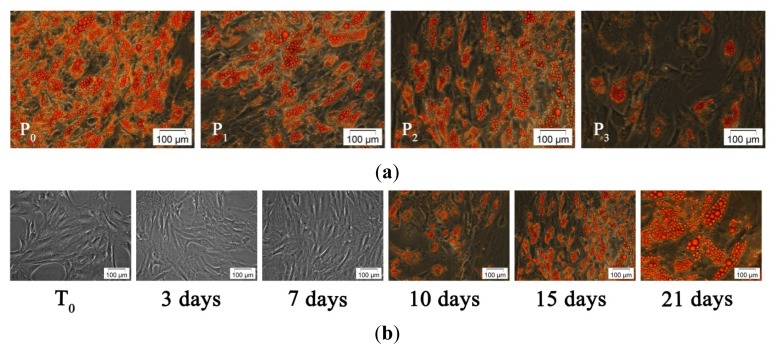
Phase contrast micrographs of (**a**) hADSCs exposed to P_0_–P_3_ for up to 15 days; (**b**) hADSCs exposed to P_2_ for up to 21 days.

Oil Red O staining of monolayers exposed to MD_0_-MD_4_ (P_0_–P_3_) over an incubation period of 15 days was used to reveal that all protocols successfully induced and sustained adipogenesis. However, significant differences between the stained samples (Figure 3a) were identified by phase contrast microscopy.

Thereby, the cell exposure to the standard medium (MD_0_) led to a fast lipid accumulation. The same results were obtained by incubating hADSCs with MD_1_ (P_1_), probably due to its chemical complexity. In contrast, P_3_ treatment resulted in a low number of differentiated cells with decreased triacylglycerol (TAG) deposits. The most balanced rate of differentiation was observed after P_2_ administration, when cells accumulated numerous small lipid droplets located around the nucleus. Taking into account the ATE possible applications, we decided to select P_2_ for further studies.

Consequently, hADSCs’ incubation with MD_1_ and MD_2_ (P_2_) for up to 21 days led to TAG accumulation starting with the tenth day after adipogenic induction. At 21 days post-adipogenic induction, cell dimensions increased several times and the morphology changed completely, from spindle to spherical shape, due to the presence of large lipid vesicles. Mature adipocytes gradually lost adherence to culture surface and floated into the medium (Figure 3b).

Rahman *et al.*[33], performed a proteomic analysis of 3T3-L1 preadipocytes subjected to adipogenesis two days *post* confluence by supplementing the growth culture medium with 0.5 mM IBMX, 0.25 μM DEX and 10 μg/mL insulin for two days and, afterwards, only with 10 μg/mL insulin until the end of the experiment. Eight days after this treatment, Oil Red O staining revealed that 90% of the cells expressed intracellular lipid accumulation. In contrast to these results, our study demonstrated that hADSCs, exposed to more complex adipogenic media (MD_1_ and MD_2_), started the TAG-accumulation at 10 days *post* induction, probably as a consequence of their more undifferentiated state compared to 3T3-L1 preadipocytes.

Aditionally, cell counting was performed at three days *post* adipogenic induction with P_2_, at the moment when MD_1_ was exchanged with MD_2_, and revealed an increase in cell number as compared to the initial moment of induction (T_0_) (data not shown). These results suggest that the composition of the media allowed cell proliferation simultaneously with hADSCs conversion to preadipocytes. These data are in accordance with phase contrast micrographs in Figure 3b, which display higher cellular density in samples exposed to P_2_ at three days *post* induction, as compared to T_0_. Moreover, in the view of prospective regenerative applications, the modulation of adipogenic conditions in order to control the equilibrium between cell proliferation and differentiation could potentially result in a greater amount of tissue formed from a set number of original cells.

**Figure 4:**
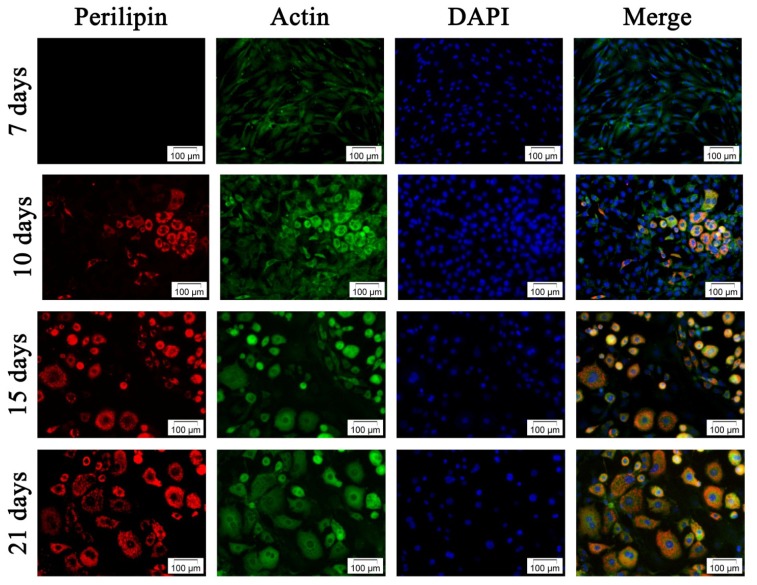
Fluorescence micrographs of the third passage hADSCs during adipogenic differentiation, stained with TRITC conjugated antiperilipin antibody (red), FITC conjugated phalloidin for actin filament labeling (green) and DAPI for highlighting the nuclei (blue).

Double fluorescence staining for perilipin and actin filaments allowed us to relate the changes in actin cytoskeleton to the adipogenic status during differentiation. Notably, perilipin fluorescence detection is correlated with the presence of intracellular lipid droplets highlighted by Oil Red O staining at 10 days *post* induction (Figure 3). *Perilipin* was not detected in undifferentiated cells and its levels of expression increased remarkably during the exposure to the adipogenic conditions ensured by P_2_ (MD_1_ and MD_2_), suggesting its high potential as an adipogenic inducer (Figure 4).

Due to the progressive TAG accumulation during adipogenesis, the cell morphology changed from spindle to spherical shape (Figure 4). Our study reveals that the increasing cell volume during adipogenesis determines the reorganization of the actin filaments. These observations are in accordance with Verstraeten *et al.*[34], who demonstrated that actin stress fibers, vimentin and microtubules reorganize into a cortical network lining membrane caves, cage-like structures surrounding lipid droplets and into a network under the plasma membrane, respectively. What is more, Kanzaki *et al.*[35] showed that insulin stimulation increased cortical actin levels, which was subsequently followed by increased actin polymerization in the perinuclear region.

##### 2.2.2.1. Quantitative RealTime RT-PCR Assay

Different stages of adipogenesis were highlighted by evaluating both the early and late adipogenic markers expression levels. *PPARγ2* and *aP2* are key regulators of the process and thus, critical adipogenic markers. *PPARγ2* is the key transcriptional regulator of adipocyte differentiation, along with C/EBPα. The *PPARγ2* isoform typically represents the *PPAR* family of transcriptional factors in adipose tissue, where, once activated, it stimulates lipid uptake and adipogenesis.

**Figure 5:**
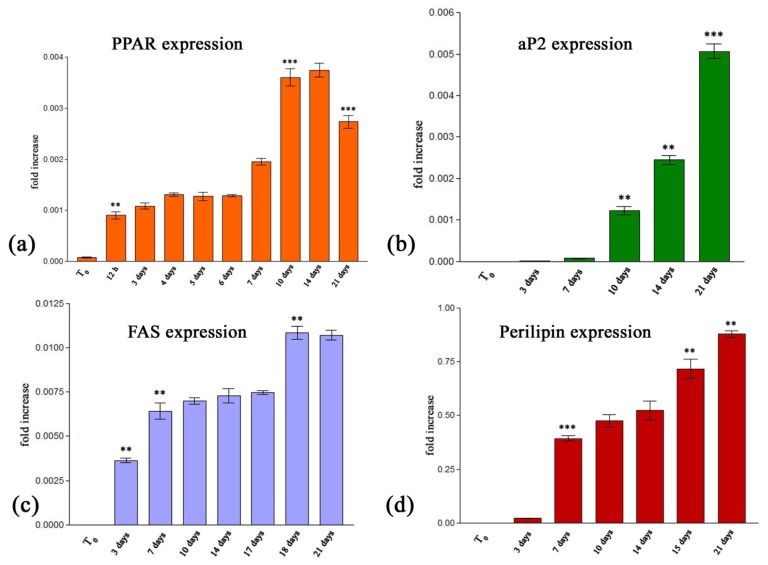
Gene expression profiles of (**a**) *PPARγ2*; (**b**) *aP2*; (**c**) *FAS* and (**d**) *perilipin* as quantified by RealTime RT-PCR.

RealTime RT-PCR results revealed a basal activity of the *PPARγ2* gene before the induction of adipogenic differentiation using P_2_ (Figure 5a).

Obvious genic activation appeared 12 h after the adipogenic cocktail was added to the cell culture (*p* < 0.01). No other significant changes in *PPARγ2* mRNA levels occurred between 12 h and seven days. A second increase in the *PPARγ2* mRNA level was revealed at day 10 *post* induction as compared to seven days (*p* < 0.001). This upregulated pattern of expression was maintained including even day 14 *post* induction. A statistical significant decrease in *PPARγ2* mRNA was detected on day 21 (*p* < 0.001) as compared to 14 days. These data confirm that *PPARγ2* is one of the first transcription factors upregulated in adipogenesis.

Our *PPARγ2* expression profile is correlated with the results reported by Saladin *et al.*[36], although Zhang *et al.*[37] showed that the protein expression of *PPARγ2* increases only in the first 24 h of the adipogenesis.

The fatty acid transporter *aP2* selectively enhances the activities of *PPARγ2* and, as a consequence, it massively relocates to the nucleus in response to *PPARγ2* selective ligands which they activate [38]. An increasing expression profile of this marker during the 21 days of induced adipogenesis was highlighted in Figure 5b. The first detection of *aP2* mRNA was possible at day 7 *post* induction. *aP2* displayed an upregulated expression profile starting with the tenthday of adipogenesis (*p* < 0.01) and the gene expression level doubled between the fourteenth and the twenty-first day of differentiation (*p* < 0.001), confirming *aP2* as late adipogenic marker.

It was reported that aP2 associates with specific nuclear membrane proteins in the presence of long-chain fatty acids or synthetic PPAR ligands [39]. Tan *et al.*[38] showed that aP2 delivers troglitazone to PPARγ2 through direct association between the binding protein and the receptor, thus highlighting the relevant use of this antidiabetic drug in the composition of MD_1_ which we proposed for protocol P_2_.

A statistical significant increase (*p* < 0.001) in *FAS* gene expression (Figure 5c) was observed during the first seven days of induced adipogenesis, compared to the T_0_ undifferentiated state. This suggests the activation of fatty acid synthesis. No other considerable changes occurred in *FAS* mRNA levels between the seventh and the seventeenth day. However, the statistically significant (*p* < 0.001) upregulated *FAS* expression pattern was detected on day 18 *post* induction and was maintained until the end of the experiment, suggesting that fatty acids continue to be synthesized inside mature adipocytes. Thus, the synthesis of TAG is required throughout the process. Our findings are in agreement with the results obtained by Clarke *et al.*[40] who showed that insulin and triiodothyronine function synergistically and had a crucial role in inducing *FAS* expression.

The late adipogenic marker *perilipin* also displayed an increasing gene expression profile (Figure 5d). Although the first detection of *perilipin* mRNA was possible on day 3 *post* induction, a statistically significant (*p* < 0.001) gene activation corresponds to day 7 of adipogenesis, compared to the T_0_ initial status. An approximately constant *perilipin* profile was recorded from day 7 to day 14 of differentiation, thus reflecting *perilipin* accumulation in the cells. Starting with day 15, *perilipin* displays an upregulated expression (*p* < 0.01) and a constant increasing profile until day 21 of induced differentiation (*p* < 0.05). Taking into account that *perilipin* expression was detected starting with the third day of the adipogenic induction, while *PPARγ2* mRNA was expressed earlier, we pointed out the role played by *PPARγ2* in *perilipin* gene activation. This finding is in accordance with Prusty *et al.*[41] who reported that *perilipin* expression is regulated in 3T3-L1 preadipocytes by *PPARγ2*.

Although cellular intermediates of mature adipocytes and stem cells are still difficult to characterize at the molecular level, two stages of adipogenesis were defined. First, adult stem cells commit the differentiation process towards the adipogenic lineage and, as a result, they are converted to preadipocytes. These progenitor cells keep the morphologic characteristics of their precursors, but differ in terms of the differentiation potential, as they have lost the ability to differentiate into other mature cell types. In the second stage of the adipogenesis, preadipocytes acquire the characteristics of the mature adipocytes. Although the composition of the adipogenic cocktail is mainly accepted by scientists, the specific experimental conditions impose the optimization of the range of the adipogenic factors triggering and sustaining the process [42]. Furthermore, the design of a differentiation protocol suitable for ATE aims to achieve the complete population of a scaffold, by allowing the implanted hADSCs to proliferate before undergoing adipogenesis. Consequently, the optimization of adipogenesis kinetics targets the delay of the process in order to allow new functional tissue formation *in situ.*

## 3. Experimental Section

### 3.1. Isolation and Culture of Primary Cells

Human subcutaneous adipose tissue was harvested from adult patients undergoing elective abdominoplasty, after obtaining their written informed consent. None of them had diabetes, severe systemic illness, or was taking medication known to affect adipose tissue metabolism. All the medical procedures were performed in compliance with the Helsinki Declaration, with the approval of the Emergency Hospital for Plastic Surgery and Burns Ethical Committee (reference No. 3076/10.06.2010).

SVF, also termed PLA [43], was separated from the fatty portions of liposuction aspirates using an adapted protocol after Gimble *et al.*[9]. Based on our observations, a set of adjustments were performed to this protocol, namely a different centrifugation speed which eliminates the risk of damaging the cells during the isolation procedure and the serum concentration provided for the cells in the first days. Briefly, LAs were washed intensely with phosphate-buffered saline (PBS) supplemented with 2% fetal bovine serum (FBS, Invitrogen, Foster City, CA, USA) and 1% antibiotic-antimycotic (ABAM, Sigma-Aldrich, Co, Steinheim, Germany) and subjected to enzymatic digestion with 0.01% type I collagenase (Gibco, Foster City, CA, USA) for 60 min at 37 °C, with intermittent shaking. Subsequently, the activity of the collagenase was neutralized by adding FBS at a final concentration of 10%. To separate the mature floating adipocytes from the SVF, the digested LA was centrifuged at 420*g*, for 10 min at 4 °C. Before cell seeding, the SVF sample was treated with erythrocyte lysis buffer (0.15 M NH_4_Cl, 0.1% KHCO_3_ and 9 μM EDTA) for 5 min at 37 °C, to remove red blood cells (Scheme Ia).

In addition, the sanguine fraction of the LAs was used to isolate multipotent cells, accordingly with the method described by Francis *et al.*[44]. Briefly, the sanguine fraction was centrifuged at 420*g*, for 10 min and the pellet was exposed to the erythrocyte lysis buffer for 5 min at 37 °C, to remove red blood cells (Scheme Ib).

After these treatments, both cell suspensions were centrifuged at 420*g* for 10 min, and the pellets were resuspended in culture medium (CM) consisting of Dulbecco’s modified Eagle’s medium (DMEM, Sigma-Aldrich, Co, Steinheim, Germany), supplemented with 3.5 g/L glucose (Sigma-Aldrich, Co, Steinheim, Germany), 1.5 g/L NaHCO_3_ (Sigma-Aldrich, Co, Steinheim, Germany) and 1% ABAM. The cells derived from the biological sources mentioned above were seeded at an initial density of 1.5 × 10^4^ cells/cm^2^ and maintained in CM supplemented with 40% FBS for 24 h, at 37 °C in a humidified atmosphere of 5% CO_2_. The next day, CM was exchanged and the FBS final concentration was adjusted to 10%.

### 3.2. Subcultivation of the Cells

The culture was propagated repeatedly after achieving 75%–80% confluence in order to purify the hADSCs population, which shows better adherent properties to plastic surfaces than hematopoietic precursor cells that are discarded during the procedure.

### 3.3. Characterization of Human Adipose Derived Stem Cells

#### 3.3.1. Phenotypic Characterization

The expression of the typical mesenchymal stem cells markers CD44, CD73 and CD90 was investigated at the transcriptional level by q-PCR, whereas their presence on cell surface was assessed via Western blotting and immunofluorescence microscopy. In addition, the same techniques were used to examine the expression of CD34 as a hematopoietic stem cell marker during successive passaging.

##### 3.3.1.1. q-PCR

Total RNA from cells was isolated with TRIzol Reagent (Invitrogen, Foster City, CA, USA) according to the manufacturer’s protocols and tested for integrity on BioAnalyzer 2100 (Agilent Technologies, Waldbronn, Germany) and purity on NanoDrop spectrophotometer. Total RNA samples extracted at different stages of adipogenesis were highly pure and intact (O.D.260/O.D.280 ratios were situated between 1.8 and 2 and RNA integrity number (RIN) scored between 8.4 and 9.9). One microgramme of total cellular RNA was reverse transcribed to corresponding cDNA using iScript cDNA Synthesis kit (BioRad, Hercules, CA, USA). Sequences of gene-specific primers used in q-PCR assay are presented in Table 3. The melting temperature optimization for these primers was carried out based on standard PCR components (GoTaq DNA Polymerase kit) provided by Promega, Madison, WI, USA via gradient PCR on a Corbett thermocycler, in 52–62 °C range of temperatures, as described in Figure 2a. The visualization of PCR products was done by electrophoresis, using a molecular weight marker of 100 bp and ethidium bromide (Roth, Karlsruhe, Germany) staining.

##### 3.3.1.2. Western Blotting

hADSCs were lysed by ultrasonication and equivalent amounts of protein (50 μg), previously determined using spectrophotometric Bradford assay, were subjected to SDS-PAGE electrophoresis in 7.5% polyacrilamyde gels. The separated proteins were then electro-blotted from the gels to nitrocellulose (NC) membranes, which were then processed using the Western Breeze Chromogenic kit (Invitrogen, Foster City, CA, USA) accordingly to the manufacturer’s protocol. The CD44 mouse antihuman, CD73 rabbit antihuman and CD90 mouse antihuman primary antibodies used to highlight the expression of target cell surface proteins were purchased from Santa-Cruz Biotechnology, Inc., Heidelberg, Germany.

##### 3.3.1.3. Microscopic Immunophenotyping of hADSCs

Subconfluent hADSCs monolayers were fixed with 4% p-formaldehyde (PFA, Sigma-Aldrich, Co., Steinheim, Germany) and blocked with 2% BSA to minimize non-specific protein-protein interactions. The cells were washed and incubated with the primary antibodies mentioned above. An additional CD34 rabbit antihuman primary antibody (Santa-Cruz Biotechnology, Inc., Heidelberg, Germany) was used in this assay. Subsequently, the samples were exposed to IgG goat antimouse—FITC and IgG goat anti-rabbit—TRITC secondary antibodies (Santa-Cruz Biotechnology, Inc., Heidelberg, Germany) and finally stained with 2 μg/mL 4′,6-diamidino-2-phenylindole (DAPI, Sigma-Aldrich, Co., Steinheim, Germany) to highlight the nuclei. Images from representative fields of each sample were acquired by means of an inverted fluorescent microscope (Olympus IX71) and Cell F image software.

#### 3.3.2. Assessment of Adipogenic Differentiation

Third passage hADSCs were exposed to four original adipogenic media (MD_1_, MD_2_, MD_3_ and MD_4_) over a 21 days period of incubation and the process was evaluated by comparison with a standard adipogenic medium (MD_0_, ECACC 811D-250, Cell Applications, San Diego, CA, USA), which served as a positive control. Cells were exposed to this standardized formula of adipogenic differentiation medium, according to the manufacturer’s instructions, since the composition of the medium was not fully provided. MD_1_-MD_4_ adipogenic media were prepared by supplementing the CM with the following adipogenic inducers: IBMX, DEX, troglitazone, insulin, biotin, indomethacine, hydrocortisone, triiodothyronine and transferrin, (all purchased from Sigma- Aldrich Co., Steinheim, Germany), as described in Table 1.

The response of hADSCs to the adipogenic conditions was characterized in terms of intracellular lipid droplets accumulation, actin filaments distribution associated with the protein levels of perilipin and gene expression profiles of *PPARγ2*, *perilipin*, *aP2* and *FAS*. These changes were monitored up to 21 days of adipogenesis in a time dependent manner. Moreover, a sample of undifferentiated hADSCs nonexposed to the adipogenic conditions (T_0_) was used in our study to serve as starting point.

##### 3.3.2.1. Staining of Lipid Droplets with Oil Red O

At 3, 7, 10, 15 and 21 days *post* induction, the monolayers were fixed with 4% PFA. After permeabilization with 2% BSA and 0.1% Triton X-100, samples were incubated with Oil Red O dye (5 mg/mL in 60% isopropanol diluted 3:2 with tap water) for 1 h at 4 °C. The visualization of the intracytoplasmatic lipid droplets accumulation was revealed by contrast-phase microscopy (Olympus IX71) and Cell Imaging Software.

##### 3.3.2.2. Fluorescent Double Staining of *Perilipin* and Actin Cytoskeleton

*Perilipin* expression and actin filaments distribution were evaluated during the process of adipogenesis by fluorescence labeling, followed by microscopic visualization. The cell monolayer was washed with PBS, fixed with 4% PFA and blocked with 2% BSA to minimize nonspecific protein-protein interactions. Then, the cells were washed again and incubated with perilipin rabbit antihuman primary antibody (Santa-Cruz Biotechnology, Inc., Heidelberg, Germany) at 4 °C overnight. Subsequently, the samples were washed twice with PBS and IgG goat antirabbit-TRITC secondary antibody (Santa-Cruz Biotechnology, Inc., Heidelberg, Germany) was added for 30 min. Actin filaments were stained by incubating the samples with FITC-conjugated phalloidin (Sigma-Aldrich, Co., Steinheim, Germany) for 1 h at 37 °C. Finally, the nuclei were stained with DAPI for 15 min at room temperature.

##### 3.3.2.3. Quantitative RealTime RT-PCR

The relative expression of *PPARγ2*, *aP2*, *perilipin* and *FAS* adipogenic markers during an incubation period of 21 days was quantified by RealTime PCR (LightCycler Fast Start DNA Master SYBR Green I, Roche, Mannheim, Germany) on a LightCycler carrousel system. For statistical interpretation, each time point selected for analysis was compared to the previous one. Early and late adipogenic markers gene expression was permanently compared to mRNA levels of the same markers from normal adipose tissue. Total RNA was isolated at different stages of adipogenesis, quality tested and reverse transcribed to cDNA as previously described (3.3.2). Gradient PCR was performed to identify the appropriate annealing temperatures for *PPARγ2*, *perilipin*, *aP2* and *FAS* primers. Sequences of these primers, along with the sequences designed for the reference gene GAPDH, are presented in Table 4.

The reactions were performed in triplicate and the levels of expression were normalized to the GAPDH housekeeping gene. The fold expression for the gene of interest was calculated using the ΔΔCt method [45].

### 3.4. Statistical Analysis

The statistical evaluation of the data was done using one-way ANOVA method followed by Bonferroni’s multiple comparison test. The results were expressed as a mean ± S.D. using GraphPad Prism Software, version 3.03 for Windows.

## 4. Conclusions

The requirements in the modern regenerative medicine applications impose a rigorous control of the cell commitment towards mature cell types and therefore the modulation of adipogenic conditions for ATE was the premise of our studies. In the context of developing an ATE successful approach, we managed to design a novel protocol to control both the conversion of undifferentiated hADSCs to adipogenic precursors and the kinetics of the mature adipocytes achievement. Our protocol accomplishes with this strategy and is based on the administration of two adipogenic media. Up to three days *post* confluence, hADSCs were exposed to a medium containing a complex adipogenic cocktail (MD_1_), while further events of the process were supported by MD_2_, which lacks IBMX, DEX and troglitazone main adipogenic inducers. Insulin was permanently included in the formulation of these media and biotine, indomethacine, hydrocortisone, T3 and transferrin were the pro-adipogenic molecules which maintained the late events of adipogenesis. This approach led us to achieve an optimum kinetics in the conversion of precursor cells into mature adipocytes, corresponding to the regenerative related applications. Our results regarding the adipogenesis evolution rate in the 2D system could provide a valuable basis for modern ATE applications.

## Figures and Tables

**Scheme I:**
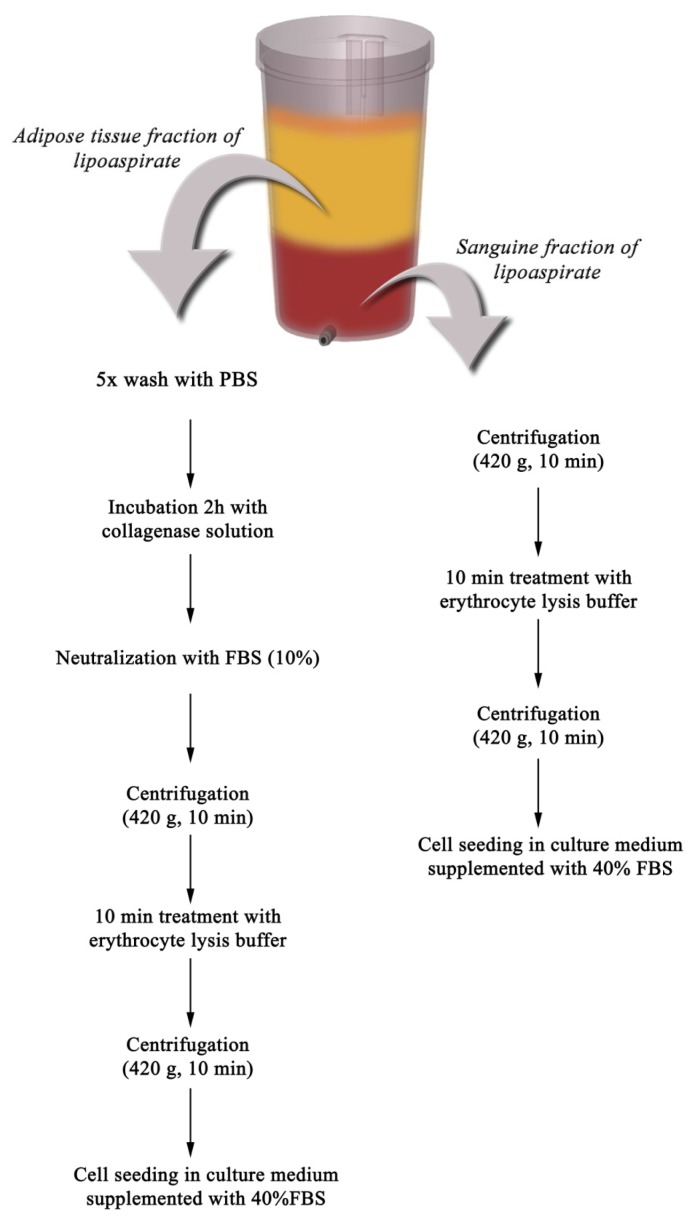
Isolation of PLA derived cells from (**a**) adipose tissue fraction; (**b**) sanguine fraction.

**Table 1:** Adipogenic media composition of P_0_–P_3_ protocols during 21 days of induced adipogenesis.

Medium	Composition	
MD_0_	Standard adipogenic differentiation medium (commercial formula code 811D-250), Cell Applications, San Diego, CA, USA	

MD_1_	CM+	0.5 mM IBMX
		1 μM DEX
		1 μg/mL Troglitazone
		1 μM Insulin
		10 μg/mL Biotin
		200 μM Indomethacine
		0.1 mM Hydrocortisone
		0.2 mM 3,3′,5-Triiodothyronine
		10 μg/mL Transferrin

MD_2_	CM+	10 μg/mL Biotin
		200 μM Indomethacine
		0.1 mM Hydrocortisone
		0.2 mM 3,3′,5-Triiodothyronine
		10 μg/mL Transferrin

MD_3_	CM+	0.5 mM IBMX
		1 μM DEX
		1 μg/mL Troglitazone
		1 μM Insulin

MD_4_	CM+	1 μM Insulin
		0.2 mM 3,3′,5-Triiodothyronine

**Table 2:** Timeline describing the administration of P_0_–P_3_ protocols during 21 days of induced adipogenesis.

	*P*_0_	*P*_1_	*P*_2_	*P*_3_
Day 1–3	MD_0_	MD_1_	MD_1_	MD_3_
Day 4–21	MD_0_	MD_1_	MD_2_	MD_4_

**Table 3:** Forward and reverse sequences of primers used to identify CD markers.

Target	Nucleotidic sequence	Fragment length
*CD44 F*	5′-CATTCAAATCCGGAAGTGCT-3′	208 bp
*CD44 R*	5′-GTTGCCAAACCACTGTTCCT-3′	
*CD73*	*F* 5′-CGCAACAATGGCACAATTAC-3′	224 bp
*CD73*	*R* 5′-CTCGACACTTGGTGCAAGA-3′	
*CD90*	*F* 5′-TGAAGGTCCTCTACTTATCCGC-3′	111 bp
*CD90*	*R* 5′-GCACTGTGACGTTCTGGGA-3′	
*CD34*	*F* 5′-CCACAGGAGAAAGGCTGGGCG-3′	157 bp
*CD34*	*R* 5′-GCCTTGCCCCACCTAGCCGA-3′	

**Table 4:** Forward and reverse sequences of primers used to identify early and late adipogenic markers.

Target	Nucleotidic sequence	Fragment length
*PPAR*γ*2 F*	5′-TTACACAATGCTGGCCTCCTT-3′	99 bp
*PPAR*γ*2*	*R* 5′-AGGCTTTCGCAGGCTCTTTAG-3′	
*aP2 F*	5′-ATGGGATGGAAAATCAACCA-3′	104 bp
*aP2 R*	5′-GTGGAAGTGACGCCTTTCAT-3′	
*Perilipin F*	5′-ATGCTTCCAGAAGACCTACA-3′	224 bp
*Perilipin R*	5′-CAGCTCAGAAGCAATCTTTT-3′	
*FAS F*	5′-GCTGGAAGTCACCTATGAAG-3′	205 bp
*FAS R*	5′-TGAAGTCGAAGAAGAAGGAG-3′	
*GAPDH F*	5′-AAGGTCGGAGTCAACGGATT-3′	224 bp
*GAPDH R*	5′-CTCCTGGAAGATGGTGATGG-3′	
